# The outcomes of modified endoscopic mucosal resection and endoscopic submucosal dissection for the treatment of rectal neuroendocrine tumors and the value of endoscopic morphology classification in endoscopic resection

**DOI:** 10.1186/s12876-020-01340-w

**Published:** 2020-06-26

**Authors:** Xiang-Yao Wang, Ning-Li Chai, En-Qiang Linghu, Shao-Tian Qiu, Long-Song Li, Jia-Le Zou, Jing-Yuan Xiang, Xing-Xing Li

**Affiliations:** Department of Gastroenterology and Hepatology, Chinese PLA General Hospital, No. 28 Fuxing Road, Beijing, 100853 China

**Keywords:** ESD, M-EMR, Rectal neuroendocrine tumors, Endoscopic morphology classification

## Abstract

**Background:**

To compare the outcomes of modified endoscopic mucosal resection (m-EMR) and endoscopic submucosal dissection (ESD) for rectal neuroendocrine tumors (NETs) and evaluate the value of endoscopic morphology classification in endoscopic resection (ER).

**Methods:**

Patients with rectal NET diameters less than 2 cm who were treated between April 2007 and January 2019 were enrolled. The endoscopic morphology of rectal NETs was classified based on the endoscopic views. Patients who underwent ESD and m-EMR were compared. Baseline characteristics as well as en bloc resection, complete resection, the procedure time, adverse events and the risk factors associated with incomplete resection were analyzed.

**Results:**

A total of 429 patients with 449 rectal NETs were enrolled for the classification of endoscopic morphology and were classified into four types (Ia, IIb, II, and III). There were 79 patients in the m-EMR group and 259 patients in the ESD group before matching. Propensity score matching created 77 pairs between the two groups that were well balanced. The mean procedure time was significantly shorter for m-EMR than for ESD (9.1 ± 4.4 min vs 16.0 ± 7.9 min, *P* = 0.000). The rates of en bloc resection (98.7% vs 100%; *P* = 1.000), complete resection (90.9% vs 93.5%, *P* = 0.548) and adverse events (2.6% vs 2.6%, *P* = 1.000) were similar between the two groups. Univariate and multivariate analyses showed that histopathological grade and endoscopic morphology were associated with incomplete resection.

**Conclusion:**

Both ESD and m-EMR are effective and safe for the treatment of rectal NETs. Endoscopic morphology should be considered along with histopathological grade for ER.

## Background

The incidence of neuroendocrine tumors (NETs) has increased in the past few decades [1]. Rectal NETs are the second most common type of digestive NET after tumors of the small intestine, and their rapidly increasing incidence has been thought to be due to the increased number of colonoscopies [2]. The prevalence of screening colonoscopies is 0.05–0.07% [3, 4]. Although rectal NETs are generally asymptomatic and indolent, metastases can occur in some patients even with relatively small tumors [5]. The prognosis of progressing rectal NETs is similar to that of rectal cancer [5, 6]; therefore, the diagnosis and treatment of early rectal NETs are of great importance.

Endoscopic resection (ER), including conventional EMR, m-EMR and ESD, has been shown to be a safe and effective modality for the treatment of small and localized early rectal NETs [7–10]. However, the outcomes of different ER techniques have been shown to vary in different studies [11, 12], and the optimal type of ER is still controversial. M-EMR was developed from EMR and includes EMR with cap (EMR-C), EMR with ligation (EMR-L), EMR with circumferential incision (EMR-CI) and EMR using a dual-channel endoscope; these have all been proven to be safe and effective methods for treating rectal NETs [13–17] and have been widely used around the world. ESD has also been reported to have higher en bloc and complete resection rates than conventional EMR [9], although ESD is slightly more complicated and time consuming than EMR and m-EMR. The optimal strategy for ER in rectal NETs still requires additional studies in order to provide strong evidence.

For the ER of rectal NETs, tumor metastasis is the first aspect to be excluded. Studies have shown that factors such as lesion size, pathological grade, lympho-vascular invasion, and atypical features are associated with metastasis [5, 10, 18, 19]. These factors should be considered in the preoperative evaluation combined with examinations such as EUS, CT, and MRI to identify muscularis invasion as well as lymph node or distant metastasis before ER. Factors associated with incomplete resection represent another aspect that should be highlighted, and these factors include lesion size [15], central depression on the surface [20] and location [21]. Of note, endoscopic appearance is related to both metastasis and incomplete resection. Thus, attention should be paid to endoscopic morphology during ER for rectal NETs, and it may be useful to classify the endoscopic morphology into different types. However, rectal NETs are a type of subepithelial tumor that differs from epithelial neoplasia [22]; because the classification of superficial neoplastic lesions of the digestive tract is not suitable for rectal NETs, endoscopic morphology should be classified according to the characteristics of rectal NETs.

In this study, the endoscopic morphology of rectal NETs was classified into types based on endoscopic characteristics. The outcomes of m-EMR and ESD were compared, and the value of endoscopic morphology classification for ER was also evaluated.

## Methods

### Patients and lesions

**Fig. 1:**
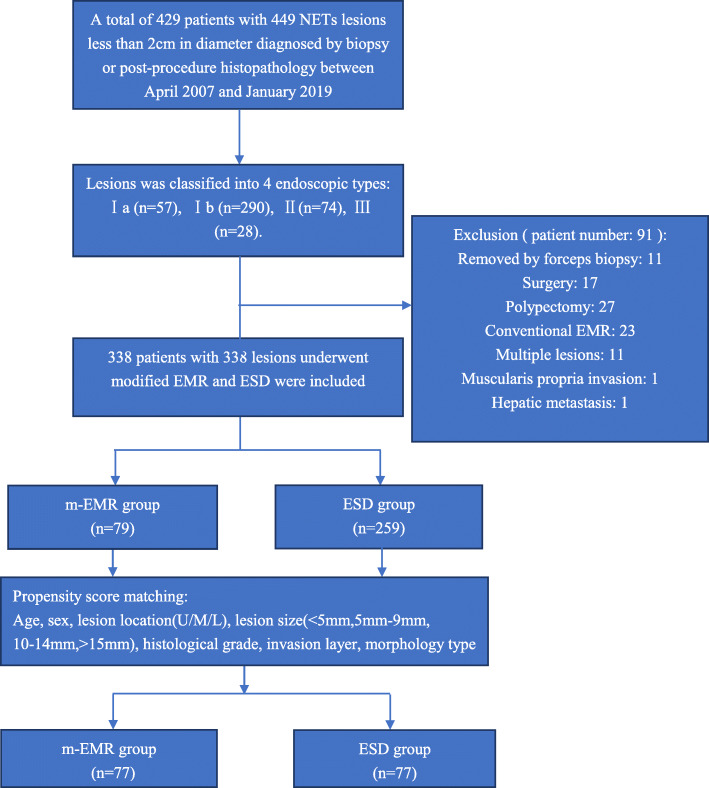
Flow chart of the study

Patients with rectal NETs less than 2 cm in diameter who underwent ER or surgery in a large tertiary, academic center from April 2007 and January 2019 were enrolled in the endoscopic morphology classification. Related data of the patients and lesions collected from our clinical and endoscopic databases were analyzed. After the analysis of endoscopic views for endoscopic morphology classification, patients and lesions other than m-EMR or ESD were excluded. The exclusion criteria were as follows: (1) tumors removed by biopsy forceps, polypectomy or surgery; (2) tumors resected by conventional EMR; (3) muscularis propria invasion or lymph node or distant metastasis confirmed before the endoscopic procedure; and (4) patients with multiple lesions. Endoscopic ultrasonography (EUS), abdominopelvic computed tomography (CT) or magnetic resonance imaging (MRI) were used to exclude muscularis invasion as well as lymph node or distant metastasis before the procedure. The flowchart is shown in Fig. 1. Written informed consent was obtained from all patients before performing any endoscopic procedures. The study was approved by the Ethics Committee of the Institutional Review Board of the Chinese PLA General Hospital.

### Endoscopic morphology types

The classification was based on the evaluation of the images in the endoscopic database. The evaluation was conducted by three endoscopists (En-Qiang Linghu, Ning-Li Chai, Xiang-Yao Wang). Two endoscopists (Ning-Li Chai, Xiang-Yao Wang) evaluated the images of all patients independently, the results were compared after evaluation. The cases with difference were re-evaluated by three endoscopists and discussed to determine the final types. The endoscopic morphology of rectal NETs was classified into four types, Ia, Ib, II and III, which were based on endoscopic characteristics with the rectal lumen adequately inflated. The angle between the edge of the tumor and the bottom of the mucosa was named θ, which was an important factor for determining the type of lesion. Other factors included elevated extension and depression as well as obvious ulcers on the surface of the tumor. The height of tumor measured by biopcy forceps can reduce the bias and facilitate to differentiate type Ib and II. The height of type II is lower than the height of the closed cups of biopsy forceps.

### Propensity score matching analysis

Propensity score matching was used to minimize the bias between the m-EMR group and the ESD group. Patient- and lesion-related characteristics were used as independent variables. Age was categorized by the median values, and lesion size was categorized by the diameter range. Finally, the matching variables were as follows: age (< 49 years, ≥49 years), sex (male, female), lesion size (< 5 mm, 5 mm–9 mm, 10–14 mm, > 15 mm), lesion location (upper third, middle third, lower third of the rectum), histological grade (grade 1, grade 2), invasion layer (mucosal, submucosal), and endoscopic morphology type (Ia, Ib, II, III). Lesions in the m-EMR group were matched with those in the ESD group at a 1:1 ratio without replacement. The match tolerance was set at 0.01.

### Outcomes

The outcomes of this study were to compare the results, including the procedure time, en bloc resection, complete resection, the complications, and the recurrence rates of m-EMR and ESD. Endoscopic morphology types were classified according to the endoscopic view of the lesion, and their values were evaluated in m-EMR and ESD procedures for the treatment of rectal NETs. Univariate and multivariate analyses were used to evaluate the risk factors associated with incomplete resection.

### M-EMR and ESD procedures

All patients with NETs were detected by colonoscopy. Preoperative EUS was performed using a UM3R ultrasonic miniprobe (UMP, 20 MHz; Olympus, Tokyo, Japan) to evaluate the tumor size and invasion depth. A single-channel endoscope (GIF-Q260J, PCF-Q260J, Olympus, Tokyo, Japan) was used for the procedures. EMR-C, EMR-CI and ESD were carried out with the use of a transparent cap on the tip of the endoscope. A band ligation device was used for EMR-L. A polypectomy snare (Cook, Winston-Salem, USA) was used to remove the tumor in the m-EMR procedure. A dual knife (Olympus, Tokyo, Japan) and/or an IT (Olympus, Tokyo, Japan) was used for the incision of the mucosa and for submucosal resection. Hemostatic forceps were used to stop and prevent bleeding during the procedure. The VIO200D electrosurgical unit (ERBE, Tubingen, Germany) was used for all the procedures.

#### EMR-C

A submucosal injection of a 1:10000 epinephrine-saline solution mixed with a small amount of methylene blue was used to provide a submucosal cushion. The lesion was then sucked into the transparent cap on the tip of the endoscope. Snaring resection was used to remove the lesion. Hemostasis using hemostatic forceps or hemostatic clips was performed if necessary.

#### EMR-L

EMR-L was firstly reported as ESMR-L by Ono A. and Fujii T. for rectal carcinoids in 2003 [23]. The tumor was aspirated into the ligation device after the submucosal injection, and then the elastic band was deployed. Snaring was carried out below the band to remove the tumor. The other steps were similar to those of EMR-C.

#### EMR-CI

A dot was marked on the circumference of the lesion, followed by submucosal injection; after that, snaring was performed effectively due to the circumferential incision. The other steps were similar to those of EMR-C.

#### ESD

**Fig. 2:**
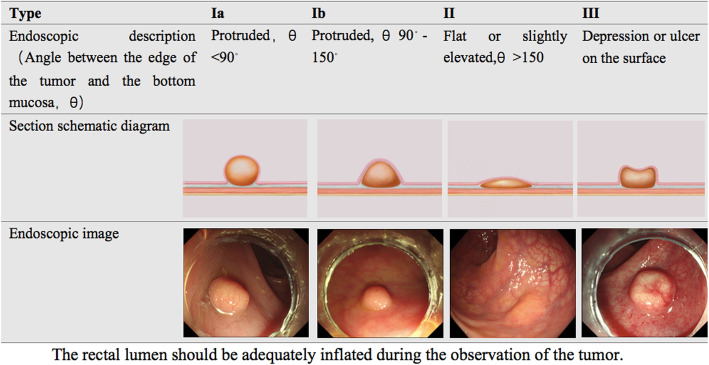
The characteristics of endoscopic types of rectal neuroendocrine tumor

In the ESD procedure, dots were marked approximately 5 mm from the periphery of the lesion. Circumferential mucosal incisions were made using a dual knife after the submucosal injection, and submucosal dissection was then carried out using the dual knife or IT knife until the tumor was completely removed. Endoscopic hemostasis was performed using hemostatic forceps to stop the bleeding during the procedure and to coagulate the exposed vessels of the defect after resection (Fig. 2).

The procedure time was measured from the submucosal injection to the completion of ER. The tumor size was measured with endoscopic biopsy forceps, EUS or pathological evaluation of the specimen.

### Histopathological evaluation

After resection, the specimens were fixed and lesion size was measured on a plate before the specimens were fixed in formalin solution. The specimens were then stained with hematoxylin & eosin (H&E) for immunohistochemistry (IHC) before the evaluation. The pathological evaluation included the size, grade, invasion layer, and lateral and vertical margins, and the evaluation was performed by a pathologist and was based on the 2010 World Health Organization classification of tumors of the digestive system [24]. Ki-67 was used to evaluate the proliferation and classify the grade of the tumor. CD-34 and D2–40 were used to evaluate the lympho-vascular invasion. Grade1 was determined as mitotic count < 2 per 10 high-power fields (HPF) and/or Ki67 ≤ 2%; Grade 2 was determined as mitotic count 2–20 per 10 HPF and/or Ki67 3–20%.

### Definition

En bloc resection was defined by the fact that the tumor was endoscopically resected in its entirely in one piece. Complete resection (R0) was defined as no evidence of a tumor on both vertical and lateral margins upon histological examination. Incomplete resection was defined by the fact that tumor-free margins were not achieved in the ER.

Complications related to the procedure included postprocedural bleeding and perforation. Postprocedural bleeding was defined as hematochezia-required endoscopic hemostasis or surgery. Perforation was defined as the defect of the whole rectal wall; the surrounding tissues or organs could be seen through the hole during the procedure.

### Follow-up

Patients were recommended to undergo regular endoscopic examinations. For patients with complete resection, a colonoscopy was scheduled for 6, 12, and 24 months after the procedure. For patients with incomplete resection but who refused to undergo additional surgery, strict follow-up colonoscopy was performed at 3, 6, and 12 months, and if there was no local recurrence, a colonoscopy was recommended once a year. If there was suspicion of recurrence during the follow-up colonoscopy, a biopsy was performed.

### Statistical analysis

Continuous variables were analyzed with t tests and Mann-Whitney U tests. The chi-square test was used for categorical data. Propensity score matching was used to match the variables between the two groups. Multivariable logistic regression analysis was performed to evaluate the factors associated with incomplete resection. A *P* value < 0.05 was considered statistically significant. All statistical analyses were performed using SPSS statistical software version 23.0 (SPSS, Inc., Chicago, IL, USA).

## Results

### Endoscopic morphology types

A total of 429 patients with NET lesions less than 2 cm in diameter were enrolled in the study of endoscopic morphology type. Type I was the most common type, including Ia and Ib, in which Ib accounted for the most frequent type. The characteristics and proportions of the four types are shown in Fig. 2 and Table 1.

**Table 1:** Baseline characteristics of patients and tumors

	Total(*n* = 338)	m-EMR(*n* = 79)	ESD(*n* = 259)	*P* value
Patient Characteristics				
Age, y				0.376
Mean ± SD	49.49 ± 10.8	50.4 ± 11.1	49.2 ± 10.7	
Median (range)	49(15–80)	49(16–77)	49(15–80)	
Sex, n (%)				0.762
Male	206(60.9%)	47(59.5%)	159 (61.4%)	
Female	132(39.1%)	32(40.5%)	100 (38.6%)	
Tumor Characteristics				
Lesion size, mm				0.004*
Mean ± SD	6.8 ± 2.9	5.8 ± 1.9	7.1 ± 3.1	
Median (range)	6.0(2–18)	6.0 (2–10)	6.0 (2–18)	
Lesion size group, n (%)				0.011*
< 10 mm	275(81.4%)	72(91.1%)	203(78.4%)	
≥ 10 mm	63(18.6%)	7(8.9%)	56(21.6%)	
Location, n(%)				
Upper	22(6.5%)	6(7.6%)	16(6.2%)	0.552
Middle	170(50.3%)	43(54.4%)	127(49.0%)	
Lower	146(43.2%)	30(38.0%)	116(44.8%)	
Histopathological grade, n (%)				0.681
Grade 1	286(84.6%)	68(86.1%)	218(84.2%)	
Grade 2	52(15.4%)	11(13.9%)	41(15.8%)	
Invasion layer, n (%)				0.636
Mucosal	75(22.2%)	16(20.3%)	59(22.8%)	
Submucosal	263(77.8%)	63(79.7%)	200(77.2%)	
Endoscopic morphology, n (%)				
Ia	39(11.5%)	6(7.6%)	33(12.7%)	0.146
Ib	230(68.1%)	59(74.7%)	171(66.0%)	
II	51(15.1%)	13(16.4%)	38(14.7%)	
III	18(5.3%)	1(1.3%)	17(6.6%)	
Outcomes				
Procedure time, min				0.000^*^
Mean ± SD	15.3 ± 9.4	9.1 ± 4.4	17.2 ± 9.7	
Median (range)	12.0 (3–56)	8.0 (3–26)	15.0(4–56)	
En bloc resection, n (%)	334(98.8%)	78 (98.7%)	256 (98.8%)	1.000
Complete resection(R0), n (%)	310(91.7%)	72(91.1%)	238(91.9%)	0.832
Procedure-related adverse events, n (%)	9(2.7%)	2(2.6%)	7(2.7%)	0.572
Postprocedural bleeding	7(2.1%)	1(1.3%)	6(2.3%)	
Perforation	2(0.6%)	1(1.3%)	1(0.4%)	
Operation involving incomplete resection^a^, n (%)	7(2.1%)	1(1.3%)	6(2.3%)	0.902

*ESD* Endoscopic submucosal dissection, *m-EMR* Modified endoscopic mucosal resection, *SD* standard deviation

*p < 0.05

^a^One patient in the m-EMR group and 4 patients in the ESD group underwent subsequent surgery, two patient in the ESD group underwent additional ESD due to the positive resection margins

### Baseline characteristics and treatment outcomes of m-EMR and ESD before propensity score matching

The results of baseline characteristics and treatment outcomes of m-EMR and ESD before propensity score matching were shown in Table 1. A total of 338 patients met the inclusion criteria: 79 patients in the m-EMR group (EMR-C 23, EMR-L 26, EMR-CI 30) and 259 patients in the ESD group. No significant differences in the baseline characteristics were found between the two groups except for tumor size. The mean tumor size in the ESD group was larger than that in the m-EMR group, and the ESD group had a larger proportion of tumors that were ≥ 10 mm in size. The procedure time was significantly different between the two groups. The m-EMR group had a shorter procedure time than the ESD group (9.1 ± 4.4 vs 17.2 ± 9.7). The rates of en bloc resection (98.8% vs 98.7%), complete resection (91.7% vs 91.1%) and adverse events (2.4% vs 2.6%) were similar between the two groups. All the patients with adverse events were successfully managed with endoscopic treatment. No significant differences in the baseline characteristics and outcomes were found between the different types of EMR methods groups (Table 2).

**Table 2:** Baseline characteristics and outcomes of m-EMR group

	EMR-C(*n* = 23)	EMR-L(*n* = 26)	EMR-CI(*n* = 30)	*P* value
Patient Characteristics				
Age, y (mean ± SD)	51.4 ± 12.6	48.6 ± 8.6	51.2 ± 11.9	0.641
Sex, male/female	12/11	19/7	16/14	0.226
Tumor Characteristics				
Lesion size, mm (mean ± SD)	6.1 ± 1.8	5.4 ± 2.0	6.0 ± 1.9	0.376
Lesion size group, n (%)				0.955
< 10 mm	21(91.3%)	24(92.3%)	27(90.0%)	
≥ 10 mm	2(8.7%)	2(7.7%)	3(10.0%)	
Location, n(%)				0.278
Upper	1(4.3%)	1(3.9%)	4(13.4%)	
Middle	12(52.2%)	18(69.2%)	13(43.3%)	
Lower	10(43.5%)	7(26.9%)	13(43.3%)	
Histopathological grade, n (%)				0.129
Grade 1	22(95.7%)	23(88.5%)	23(76.7%)	
Grade 2	1(4.3%)	3(11.5%)	7(23.3%)	
Invasion layer, n (%)				0.691
Mucosal	6(26.1%)	5(19.2%)	5(16.7%)	
Submucosal	17(73.9%)	21(80.8%)	25(83.3%)	
Endoscopic morphology, n (%)				0.356
Ia	0(0.0%)	2(7.7%)	4(13.3%)	
Ib	16(69.6%)	20(76.9%)	22(76.7%)	
II	6(26.1%)	4(15.4%)	3 (10.0%)	
III	1(4.3%)	0(0.0%)	0(0.0%)	
Outcomes				
Procedure time, min (mean ± SD)	9.6 ± 4.8	8.5 ± 5.1	9.2 ± 3.5	0.681
En bloc resection, n (%)	23(100%)	26(100%)	29 (96.7%)	0.437
Complete resection(R0), n (%)	19(82.6%)	26(100%)	27(90.0%)	0.098
Procedure-related adverse events, n (%)	1(4.3%)	0(0.0%)	1(3.3%)	0.747
Operation involving incomplete resection, n (%)	1(4.3%)	0(0.0%)	0(0.0%)	0.291

*M-EMR* modified endoscopic mucosal resection, *EMR-C* endoscopic mucosal resection with cap, *EMR-L* endoscopic mucosal resection with ligation, *EMR-CI* endoscopic mucosal resection with circumferential incision, *SD* standard deviation

*p* < 0.05

### Baseline characteristics and treatment outcomes after propensity score matching

Seventy-seven pairs were matched by propensity score matching. The two groups were well balanced. The baseline characteristics showed no significant difference between the two groups. The procedure time was significantly different between the two groups; the m-EMR group had a shorter procedure time than the ESD group (9.1 ± 4.4 vs 16.0 ± 7.9, *P* = 0.000), which was the same as the result before propensity score matching. The other treatment outcomes, including en bloc resection (98.7% vs 100%), complete resection (90.9% vs 93.5%), and adverse events (2.6% vs 2.6%), were not significantly different between the two groups (Table 3).

**Table 3:** Matching factors between m-EMR and ESD group and outcomes after propensity score matching

	m-EMR(*n* = 77)	ESD(*n* = 77)	*P* value
Variables matched between groups			
Patient-related variables			
Age, y (mean ± SD)	50.3 ± 11.2	50.6 ± 10.6	0.883
Sex, male/female	46/31	44/33	0.744
Lesion-related variables			
Lesion size, mm (mean ± SD)	5.9 ± 1.9	6.3 ± 2.4	0.280
Lesion size group, n (%)			0.316
< 10 mm	70(90.9%)	66 (85.7%)	
≥ 10 mm	7(9.1%)	11(14.3%)	
Location, (U / Middle / L)	5/42/30	6/43/28	0.918
Histopathological grade(G1/G2)	66/11	70/7	0.316
Invasion layer, (M/SM)	16/61	14/63	0.684
Endoscopic morphology, n (%)			0.602
Ia	5(6.5%)	5(6.5%)	
Ib	58(75.3%)	52(67.5%)	
II	13(16.9%)	17(22.1%)	
III	1(1.3%)	3(3.9%)	
Outcomes			
Procedure time, min			0.000*
Mean ± SD	9.1 ± 4.4	16.0 ± 7.9	
Median (range)	8(3–26)	14(6–40)	
En bloc resection, n (%)	76(98.7%)	77(100%)	1.000
Complete resection(R0), n (%)	70(90.9%)	72(93.5%)	0.548
Procedure-related adverse events, n (%)	2(2.6%)	2(2.6%)	1.000
Operation involving incomplete resection^a^, no.(%)	1(1.3%)	2(2.6%)	1.000

*ESD* Endoscopic submucosal dissection, *m-EMR* Modified endoscopic mucosal resection, *SD* standard deviation, *U* upper third of rectum, *L* lower third of rectum, *M* mucosal, *SM* submucosal

*p < 0.05

^a^There are one patient in each group underwent subsequent surgery and one patient in the ESD group underwent additional ESD due to positive resection margins

### Factors associated with incomplete resection

An analysis of factors associated with incomplete resection was performed. Univariate and multivariate analyses before propensity score matching showed that histopathological grade 2 (OR 3.478, 95%CI 1.375–8.839, *P* = 0.009) as well as endoscopic morphology type II (OR 6.651, 95%CI 1.238–35.743, *P* = 0.027) and type III (OR 6.806, 95%CI 1.064–43.560, *P* = 0.043) were associated with incomplete resection (Tables 4 and 5). The univariate and multivariate analyses after propensity score matching further confirmed the results (Tables 6 and 7).

**Table 4:** Factors associated with incomplete resection before propensity score matching

	Complete resection (*n* = 310)	incomplete resection (*n* = 28)	*P* value
Patient-related variables			
Age, y (mean ± SD)	49.2 ± 10.8	52.3 ± 10.8	0.158
Sex, male/female	180/121	17/11	0.979
Lesion-related variables			
Lesion size group, n (%)			0.055
< 10 mm	256(82.6%)	19 (67.9%)	
≥ 10 mm	54(17.4%)	9(32.1%)	
Location, (U / Middle / L)	18/160/132	4/10/14	0.108
Histopathological grade(G1/G2)	267/43	19/9	0.022*
Invasion layer, (M/SM)	72/238	3/25	0.127
Endoscopic morphology, n (%)			0.004*
Ia	37(11.9%)	2(7.1%)	
Ib	217(70.0%)	13(46.4%)	
II	42(13.6%)	9(32.2%)	
III	14(4.5%)	4(14.3%)	
Outcomes variables			
Procedure type, n (%)			0.832
m-EMR	72(23.2%)	7(25.0%)	
ESD	238(76.8%)	21(75.0%)	
En bloc resection, n (%)	307(99.0%)	27(96.4%)	0.785

*ESD* Endoscopic submucosal dissection, *m-EMR* Modified endoscopic mucosal resection, *SD* standard deviation, *U* upper third of rectum, *L* lower third of rectum, *M* mucosal, *SM* submucosal

*p < 0.05

**Table 5:** Multivariate analysis for incomplete resection before propensity score matching

Variables	Odds ratio	95% CI	*P* value
Histopathological grade			
Grade 1	1(reference)		
Grade 2	3.478	1.375–8.839	0.009*
Endoscopic morphology			
Ia	1(reference)		
Ib	1.750	0.356–8.588	0.491
II	6.651	1.238–35.743	0.027*
III	6.806	1.064–43.560	0.043*

*CI* confidence interval

*p < 0.05

**Table 6:** Factors associated with incomplete resection after propensity score matching

	Complete resection (*n* = 142)	incomplete resection (*n* = 12)	*P* value
Patient-related variables			
Age, y (mean ± SD)	50.7 ± 11.0	47.6 ± 8.4	0.257
Sex, male/female	81/61	9/3	0.364
Lesion-related variables			
Lesion size, mm (mean ± SD)	6.1 ± 2.2	6.5 ± 1.6	0.318
Lesion size group, n (%)			
< 10 mm	124(87.3%)	12 (100%)	0.398
≥ 10 mm	18(12.7%)	0(0.0%)	
Location, (U / Middle / L)	9/79/54	2/6/4	0.410
Histopathological grade(G1/G2)	128/14	8/4	0.050
Invasion layer, (M/SM)	29/113	1/11	0.525
Endoscopic morphology, n (%)			0.022*
Ia	10(7.1%)	0(0.0%)	
Ib	104(73.2%)	6(50.0%)	
II	26(18.3%)	4(33.3%)	
III	2(1.4%)	2(16.7%)	
Outcomes variables			
Procedure type, n (%)			0.548
m-EMR	70(49.3%)	7(58.3%)	
ESD	72(50.7%)	5(41.7%)	
En bloc resection, n (%)	141(99.3%)	12(100%)	0.922

*ESD* Endoscopic submucosal dissection, *m-EMR* Modified endoscopic mucosal resection, *SD* standard deviation, *U* upper third of rectum, *L* lower third of rectum, *M* mucosal, *SM* submucosal

*p < 0.05

**Table 7:** Multivariate analysis for incomplete resection after propensity score matching

Variables	Odds ratio	95% CI	*P* value
Histopathological grade			
Grade 1	1(reference)		
Grade 2	5.749	1.420–23.271	0.014*
Endoscopic morphology			
Ia + Ib	1(reference)		
II + III	3.896	1.212–12.528	0.022*

*CI* confidence interval

*p < 0.05

### Follow-up results

The median follow-up was 39 (range 7–137) months. One patient in the ESD group experienced recurrence 1 year after the procedure. The other patients had no recurrence or metastasis during the follow-up period. The recurrence case was a 30-year old male. He was accidentally found with a 1 cm tumor during colonoscopy. The ESD procedure for the patient achieved en-bloc resection and complete resection. The histopathological evaluation(H&E and IHC) reported that the pathological grade was G1 and no tumor cells were found in the lateral and vertical margin. However, the follow-up colonoscopy found a 4 mm elevated lesion at the ESD scar which was confirmed as G1 NETs by endoscopic biopcy. The patient refused to receive additional endoscopic resection but chose to receive regular endoscopic surveillance.

## Discussion

This study with a relatively large patient number performed at an academic, tertiary center showed that m-EMR and ESD were safe and effective treatments for rectal NETs. Histopathological grade and endoscopic morphology were factors associated with incomplete endoscopic resection. The classification of endoscopic morphology may be helpful for the choice of optimal treatment for rectal NETs.

Rectal NETs represent a type of subepithelial tumor that are usually located in the submucosal layer; most NETs are detected at an early stage. Compared with other subepithelial tumors, rectal NETs have their own characteristics. Therefore, the classification of superficial neoplastic lesions of the digestive tract is not suitable for rectal NETs, and endoscopic morphology should be classified according to the characteristics of rectal NETs. Few studies have focused on the classification of the endoscopic morphology of subepithelial tumors. Based on endoscopic characteristics, we classified the endoscopic morphology of rectal NETs into four types. The results showed that types II and III were associated with incomplete resection. Therefore, the classification may help to establish an optimal management strategy for different types (size, pathological grade, endoscopic morphology classification) of rectal NETs. Advanced ER techniques should be considered for lesions with more than one risk factor related to incomplete resection. Further studies are needed to explore more effective ER techniques, such as the endoscopic tunnel technique [25, 26] and endoscopic full-thickness resection (EFR) [27, 28], for the treatment of rectal NETs.

For rectal NETs with diameters larger than 20 mm, there is a high risk of lymph node and distant metastases [5, 29, 30]; this was the case for all the tumors in this study that had diameters less than 20 mm. ER has been proven to be an effective and safe treatment for rectal NETs without muscularis invasion and metastasis for smaller tumors [7]. The main types of ER include conventional EMR, m-EMR and ESD. ESD and m-EMR, which include EMR-C, EMR-L and EMR-CI, have been widely used in the treatment of rectal NETs and have been reported to have a high complete resection rate [11, 12]. This study compared the outcomes of m-EMR and ESD, and the results showed that both m-EMR and ESD were effective and safe treatments for rectal NETs. M-EMR had a shorter procedure time than ESD in terms of removal of the lesion, but the two procedures demonstrated similar rates of complete resection, en bloc resection and adverse events both before and after propensity score matching. However, the mean lesion size in the m-EMR group was smaller than that in the ESD group before propensity score matching, which may be due to the constricted diameter of the assisting plastic cap and ligation device in the m-EMR procedure. In this study, 63 (18.6%) patients had a lesion size ≥10 mm; 56 of these patients were treated with ESD, and they achieved a high rate of en bloc resection and complete resection. However, ER for treating tumor with size between 1 and 2 cm is still in controversy. The NANETS Consensus Guidelines for the Diagnosis and Management of Gastrointestinal Neuroendocrine Tumors [31] and ENETS Consensus Guidelines Update for Colorectal Neuroendocrine Neoplasms [32] recommended ER for the management of small rectal NETs(< 1 cm). But NCCN guideline for neuroendocrine tumors [33] recommended that ER can be used for tumor ≤2 cm after radiological assessment. Thus, more data are needed to confirm the efficacy and safety of ER for rectal NETs with diameter between 1 and 2 cm.

Because of the potential for malignancy, complete resection is important for ER. Thus, factors impacting complete resection should be explored. Studies have shown that lesion size, central depression and location are associated with incomplete resection [15, 20, 21]. The results of this study confirmed that pathological grade and endoscopic morphology type were associated with incomplete resection. The endoscopic morphology type has been reported in previous research, but it has not been further studied.

The study has some limitations. First, it was a retrospective study that was conducted in a single tertiary center, which may have the drawback of selection bias. To compensate for this, propensity score matching was used to minimize the bias and to balance the two groups. Second, the number of patient in this study was still not large enough, and further studies are required to validate the endoscopic morphology classification for the treatment of rectal NETs. Third, the follow-up was not strictly standardized, and the follow-up period in some patients was insufficient, especially in patients with incomplete resection. A long-term follow-up study is needed to verify the prognosis of rectal NET patients with incomplete ER.

## Conclusion

In conclusion, both ESD and m-EMR are effective and safe for treating rectal NETs. Endoscopic morphology should be considered an important factor along with histopathological grade for the ER of rectal NETs. ESD and m-EMR can be selectively used for different types of rectal NETs.

## Data Availability

The datasets used and/or analyzed during the current study available from the corresponding author on reasonable request.

## Abbreviations

M-EMR: Modified endoscopic mucosal resection
ESD: Endoscopic submucosal dissection
ER: Endoscopic resection
NETs: Neuroendocrine tumors
EUS: Endoscopic ultrasonography
CT: Computed tomography
MRI: Magnetic resonance imaging
